# Areca palm velarivirus 1 encoded CP suppresses antiviral RNA silencing by mediating the autophagic degradation of SGS3 and disrupting the SGS3–RDR6 interaction

**DOI:** 10.1007/s44154-025-00279-w

**Published:** 2026-01-04

**Authors:** Jiawei Wen, Huili Li, Zemu Li, Hongxing Wang, Xianmei Cao, Xi Huang

**Affiliations:** National Key Laboratory for Tropical Crop Breeding, School of Breeding and Multiplication (Sanya Institute of Breeding and Multiplication), Hainan University, Sanya Hainan, 572025 China

**Keywords:** Areca palm velarivirus 1, Autophagy, CP, SGS3, RDR6

## Abstract

Areca palm velarivirus 1 (APV1) is the causative agent of yellow leaf disease (YLD), leading to severe yield losses in areca palms. However, how APV1 counteracts host immunity remains largely underexplored, and the underlying mechanisms are still poorly understood. RNA silencing is an evolutionarily conserved antiviral defense mechanism in eukaryotes. In this study, we identify the APV1-encoded capsid protein (CP) as a viral suppressor of RNA silencing (VSR) that inhibits both local and systemic silencing triggered by single-stranded RNA (ssRNA). Mechanistically, CP interacts with host Suppressor of Gene Silencing 3 (AcSGS3), a key component of the RNA silencing pathway, and promotes its degradation via autophagy. Additionally, CP disrupts the SGS3–AcRDR6 (RNA-dependent RNA polymerase 6) interaction, impairing the RNAi signaling cascade. Our findings reveal a novel dual mechanism to counteract host RNA silencing in which APV1 CP disrupts the SGS3–AcRDR6 complex and exploits the autophagic pathway to degrade AcSGS3, thereby undermining host antiviral defenses.

**Supplementary Information:**

The online version contains supplementary material available at 10.1007/s44154-025-00279-w.

## Introduction

RNA silencing is an evolutionarily conserved mechanism of sequence-specific gene regulation that plays essential roles in growth, development, genome integrity, and stress adaptation in eukaryotes (Zhao and Guo 2022). The pathway is typically initiated by double-stranded RNA (dsRNA), or hairpin-structured RNA, or microRNA, which is cleaved into 21–24 nucleotide small interfering RNAs (siRNAs) by RNase III enzymes such as DICER in animals and DICER-LIKE proteins (DCL) in plants (Lopez-Gomollon and Baulcombe 2022; Csorba et al. 2015). These siRNAs are then loaded into ARGONAUTE (AGO) proteins to form the RNA-induced silencing complex (RISC), which guides the sequence-specific degradation or translational repression of complementary target transcripts. Viral RNAs are often targeted because they form double-stranded RNA intermediates during replication or contain extensive secondary structures that trigger the production of viral-derived siRNAs. RNA silencing is thus a conserved immune weapon triggered by the infecting virus. To amplify the silencing signal, host RNA-dependent RNA polymerases (RDRs) synthesize secondary dsRNAs, leading to the production of additional siRNAs. In plants, the Suppressor of Gene Silencing 3 (SGS3) interacts with RDR6 to form the SGS3/RDR6 complex, a crucial module that enhances RNA silencing and promotes robust, systemic antiviral defense (Iwakawa and Tomari 2022).

As a major antiviral mechanism, RNA silencing is frequently targeted by viral pathogens. Numerous plant viruses encode viral suppressors of RNA silencing (VSRs) that interfere with the pathway through diverse strategies, including siRNA binding, dsRNA sequestration, or degradation of key host silencing factors (Liu et al. 2023; Choi et al. 2023; Li et al. 2019). Notably, SGS3 is a common target of VSRs; for instance, the V2 protein of Tomato yellow leaf curl virus (TYLCV), the P2 protein of Rice stripe virus (RSV), and the coat protein (CP) of Turnip crinkle virus (TCV) have all been shown to interact with and inhibit SGS3 function (Glick et al. 2008; Du et al. 2011; Liu et al. 2023). Other VSRs, including TGBp1 of Plantago asiatica mosaic virus (PlAMV) and AC4 of Cassava mosaic virus (CMG), disrupt both SGS3 and RDR6, underscoring the central role of the SGS3–RDR6 complex in plant antiviral immunity (Liu et al. 2025; Mann et al. 2016; Okano et al. 2014). Moreover, the geminiviral βC1 satellite protein promotes the degradation of NbSGS3 via a host calmodulin-like protein (NbCaM), illustrating the continuous evolutionary arms race between plants and viruses (Li et al. 2014, 2017).

Autophagy, a conserved eukaryotic degradation pathway, also participates in antiviral defense. By delivering damaged cellular components or invasive pathogens to the vacuole for degradation, autophagy contributes to plant stress responses and immune regulation (Marshall and Vierstra 2018). Selective autophagy depends on specific receptor proteins that recognize cargo and tether it to autophagy-related protein 8 (ATG8) that facilitates autophagosome formation (Johansen and Lamark 2020; Noda et al. 2010). Recent evidence indicates that plants can exploit autophagy to restrict viral infection (Haxim et al. 2017). Conversely, viruses have evolved strategies to disrupt or manipulate host autophagy, highlighting the complex interplay between autophagy and viral pathogenesis (Wu et al. 2021, 2023).

Areca palm velarivirus 1 (APV1), a member of the genus *Velarivirus* in the family *Closteroviridae*, has been identified as the causal agent of areca palm yellowing disease (YLD), a destructive condition affecting areca palm cultivation (Wang et al. 2020, Zhang et al. 2022, Zhao et al*. *2024). APV1 possesses a large genome of approximately 17.5 kb that encodes 11 putative proteins (Cao et al. 2021), though the functions of most APV1-encoded proteins remain poorly characterized. Recent evidence suggests that the interaction between the CP and minor coat protein (CPm) of APV1 may contribute to symptom development (Meng et al. 2025). One closterovirus protein, Citrus tristeza virus (CTV) encoded p20 was recently reported to suppress antiviral RNA silencing by co-opting ATG8 to mediate the autophagic degradation of SGS3 (Zhang et al. 2025). However, the mechanisms by which APV1 counteracts host immunity are still largely unknown, and its pathogenesis remains underexplored. To investigate whether APV1-encoded proteins act as VSRs and/or interfere with host autophagy, we systematically analyzed the function of APV1 CP. Here, we demonstrate that CP acts as a strong suppressor of both local and systemic RNA silencing. Mechanistically, CP interacts with SGS3 and promotes its degradation via the autophagy pathway. Furthermore, CP disrupts the SGS3–RDR6 complex and attenuates host RNA silencing signaling. Our study uncovers a novel viral strategy in which APV1 hijacks the host autophagy machinery to degrade a key host component of the RNA silencing apparatus, thereby facilitating viral infection.

## Results

### CP suppresses local and systemic RNA silencing

**Fig. 1:**
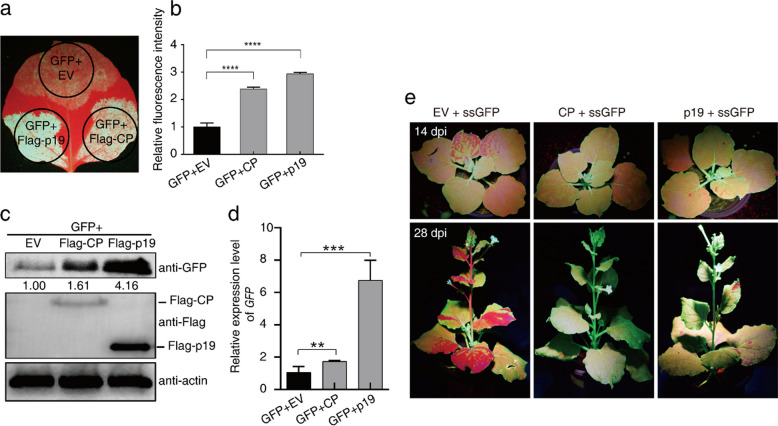
APV1 CP suppresses local RNA silencing in *N. benthamiana* plants and systemic RNA silencing in *N. benthamiana* 16c plants. **a-d** CP suppresses local RNA silencing induced by GFP mRNA in wild-type *N. benthamiana*. Three patches in the same single leaf were co-infiltrated with an Agrobacterium culture carrying a GFP-expressing construct (p35S-GFP) and the empty vector (EV), p35S-CP, or p35S-p19^TBSV^, respectively, then monitored and photographed at 3 dpi under strong blue light (**a**), and the fluorescence intensity was calculated (**b**). The infiltrated patches were sampled at 3 dpi for the quantification of GFP protein by Western-Blot (**c**) and mRNA levels by RT-PCR. The expression level of *NbActin* was used as normalizer (**d**). **e** CP suppresses systemic RNA silencing triggered by GFP mRNA (single-stranded GFP transcripts, ssGFP) in 16c plants. Images of the upper panel and the lower panel were taken for representative plants at 14 dpi and 28 dpi, respectively

To determine whether APV1 CP possesses viral suppressor of RNA silencing (VSR) activity, *GFP* was transiently co-expressed with *CP* in *N. benthamiana* leaves via agroinfiltration. As shown in Fig. 1a, GFP fluorescence was weak when expressed alone, but co-expression with either CP or the well-characterized VSR p19^TBSV^ encoded by Tomato bushy stunt virus (TBSV) significantly enhanced GFP fluorescence (Fig. 1a). Western blot and RT-PCR analysis confirmed that CP substantially increased both GFP protein and *GFP* mRNA accumulation (Fig. 1b-c), indicating that CP effectively suppresses local RNA silencing.

To further assess CP’s ability to inhibit systemic RNA silencing, we performed an agroinfiltration assay in GFP-transgenic *N. benthamiana* line 16c. Co-expression of *GFP* with an empty vector (EV) in lower leaves triggered systemic silencing in upper leaves by 10 dpi, with nearly complete silencing by 31 dpi. In contrast, co-expression of *GFP* with either *CP* or *p19*^*TBSV*^ prevented systemic silencing, as evidenced by sustained GFP fluorescence in upper leaves (Fig. 1d). Collectively, these results demonstrate that APV1 CP functions as a potent VSR, capable of suppressing both local and systemic RNA silencing.

### CP interacts with SGS3 to facilitate silencing suppression

**Fig. 2:**
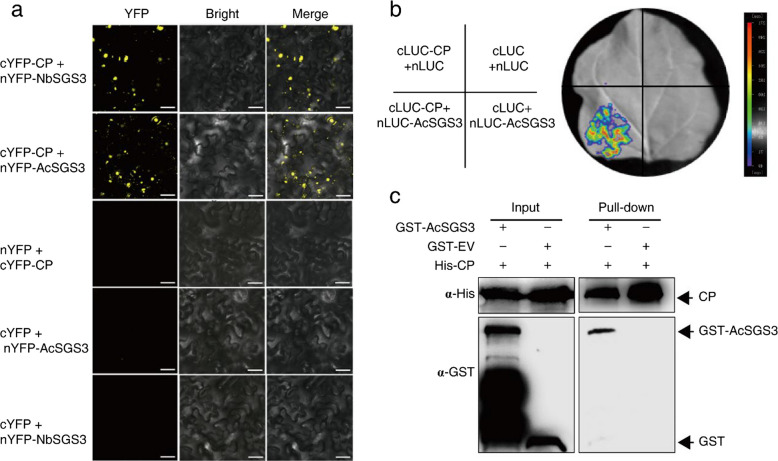
CP interacts with NbSGS3 and AcSGS3. **a** Interaction relationships of CP-AcSGS3 and CP-NbSGS3 identified by bimolecular fluorescence complementation (BiFC). YN (N-terminal YFP) and YC (C-terminal YFP) were the original empty vectors that served as the negative controls. YFP fluorescence was documented by confocal microscopy at 3 dpi. Scale bar = 50 µm. **b** Luciferase complementation assay (LCA) confirmed the interaction between CP and AcSGS3. The representative image was taken at 2 dpi. cLUC (C-terminal luciferase) and nLUC (N-terminal luciferase) were the original empty vectors that served as the negative controls. **c** Pull‐down assay detected binding between CP and AcSGS3 in vitro. Recombinant GST-AcSGS3 was incubated with His-CP. Protein samples before and after the GST pull‐downs were subjected to immunoblotting with anti‐his and anti-GST antibody, respectively

To elucidate the molecular mechanism underlying CP-mediated RNA silencing suppression, we examined its potential interaction with SGS3, a key component of the RNA silencing pathway. Using bimolecular fluorescence complementation (BiFC) assays, we observed strong YFP fluorescence when co-expression of either nYFP-NbSGS3 (*N. benthamiana* SGS3) or nYFP-AcSGS3 (areca palm SGS3, GenBank PQ496828) with cYFP-tagged CP in leaf epidermal cells (Fig. 2a). The AcSGS3-CP interaction was further verified by two independent approaches: Luciferase complementation assay (LCA) (Fig. 2b) and Pull-down assays (Fig. 2c). Subcellular localization analysis indicated the of fusion proteins RFP-CP and GFP-AcSGS3 co-localized in epidermal cells of *N. benthamiana* (Fig. S1a). Furthermore, the BiFC fluorescence of nYFP-AcSGS3 and cYFP-CP was punctiform, which was not overlapped with nucleus marker Gw5-RFP, but partially with Ghd7-RFP markers (Fig. S1b). These findings collectively establish that CP interacts with both NbSGS3 and AcSGS3, suggesting a conserved targeting strategy to subvert RNA silencing.

### Mapping the interaction domains between CP and AcSGS3

To delineate the interaction interface between APV1 CP and AcSGS3, we systematically dissected AcSGS3 into four structural domains based on bioinformatic predictions: the N-terminal domain (AcNTD, aa 1–257), zinc finger domain (AcZF, aa 258–328), XS domain (AcXS, aa 329–535; containing the conserved rice gene X and SGS3 motifs), and C-terminal coiled-coil domain (AcCC, aa 536–659). BiFC assays revealed distinct subcellular localization patterns for these interactions: while AcNTD and AcCC interactions with CP occurred primarily at the cell cytoplasm, AcZF and AcXS interactions exhibited dual nuclear and cytoplasm localization (Fig. S2a–b). Pull-down assays further confirmed that all four domains physically associate with CP in vitro (Fig. S2c–f), indicating multiple interaction sites within AcSGS3.

To identify reciprocal interaction domains in CP, we generated three truncation mutants: an N-terminal fragment (cYFP-CP1–110), a middle domain (cYFP-CP111–205), and a C-terminal fragment (cYFP-CP206–298). All mutants reconstituted YFP fluorescence when co-expressed with full-length AcSGS3, demonstrating their binding capacity. Notably, interactions involving CP1–110 or CP206–298 localized predominantly to the cytoplasm, whereas CP111–205 formed irregular punctate structures in the cytoplasm (Fig. S3). Qualitative analysis of fluorescence intensity indicated that the N- and C-terminal regions of CP mediated stronger interactions with AcSGS3 compared to the middle domain. These results indicate that CP and AcSGS3 engage through multiple interaction interfaces, with the N- and C-terminal domains of CP serving as primary binding sites, while AcSGS3 utilizes distributed domains for complex formation.

### CP induces autophagic degradation of AcSGS3

**Fig. 3:**
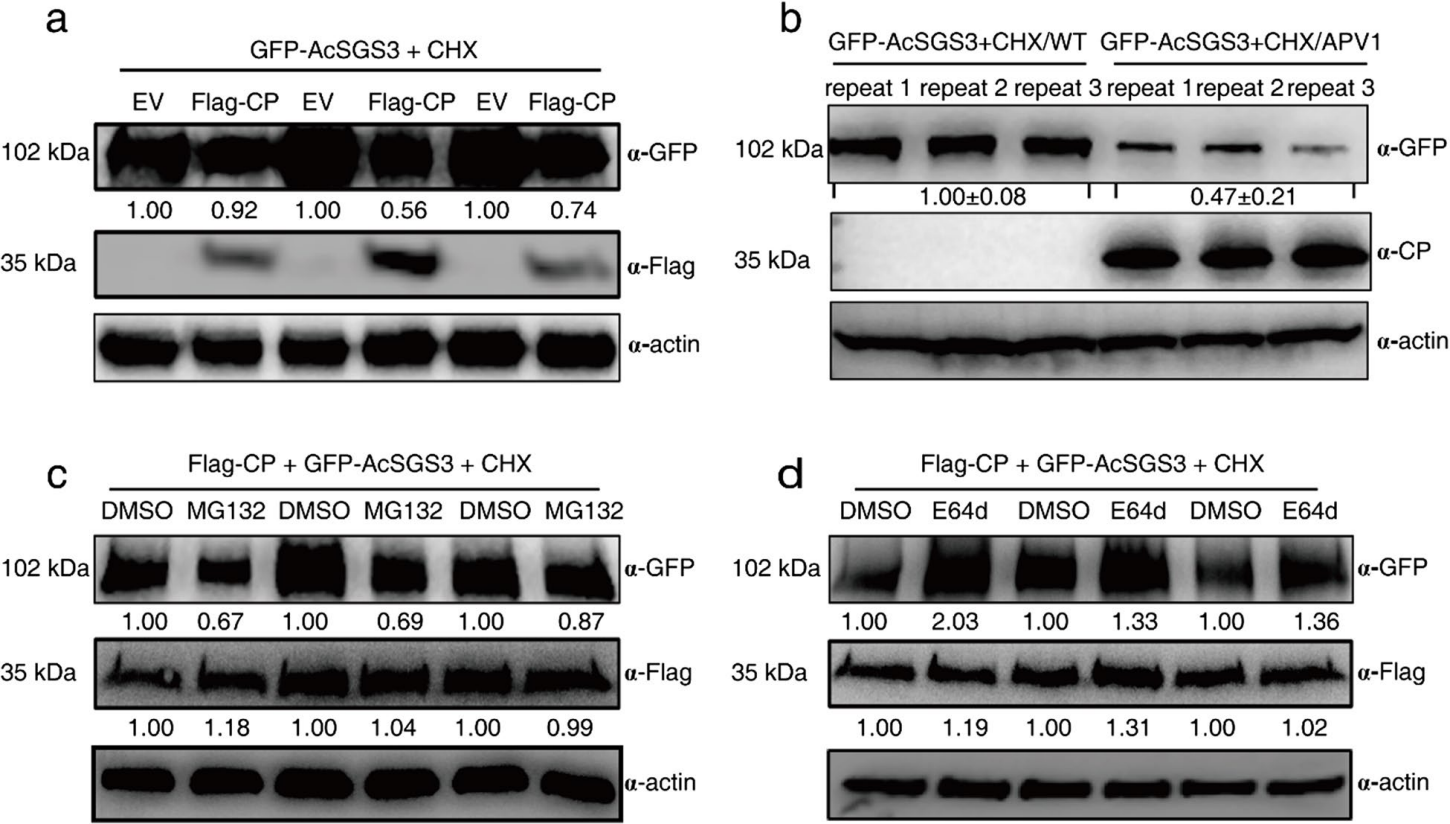
CP mediates the autophagic degradation of AcSGS3. **a-b** APV1 infection or transient expression of CP induces the degradation of AcSGS3. The accumulation level of AcSGS3 decreased in the presence of CP. GFP-AcSGS3 was transiently co-expressed with Flag-CP or Flag (the empty vector, EV) in *N. benthamiana* leaves (**a**). GFP-AcSGS3 was transiently expressed in APV1-infected *N. benthamiana* or health control (WT) (**b**). Total protein was extracted at 3 dpi and antibodies against GFP or Flag were used as a primary antibody. Actin was used as loading controls. **c-d** Effect of the autophagy inhibitor and proteasome inhibitor on AcSGS3 stability. Flag-CP and GFP-AcSGS3 were transiently co-expressed in *N. benthamiana* leaves by agroinfiltration. At 48 h post inoculation (hpi), the infiltrated leaves were treated with a protein synthesis inhibitor cycloheximide (CHX) with MG132 (**c**) or with E64D (**d**) for an additional 12 h then sampled for Western blot analyses. DMSO was used as the negative control

To further investigate whether CP functions as a VSR through its interaction with AcSGS3, we examined the effect of CP on AcSGS3 protein stability. Transient co-expression assays showed that Flag-tagged CP significantly reduced the accumulation of GFP-AcSGS3 compared with the empty vector control (Fig. 3a), suggesting CP-mediated degradation of AcSGS3. To further determine whether APV1 infection causes SGS3 degradation, GFP-AcSGS3 was transiently expressed in APV1-infected *N. benthamiana*. Western blot analysis revealed that the accumulation of GFP-AcSGS3 was sharply reduced to 38% of the control level (Fig. 3b), indicating that either APV1 infection or transient expression of CP induces the degradation of AcSGS3. To identify the pathway responsible for AcSGS3 degradation, we used specific inhibitors targeting the two major eukaryotic protein degradation systems. Treatment with the autophagy inhibitor E64D substantially restored GFP-AcSGS3 levels, whereas the proteasome inhibitor MG132 had no effect (Fig. 3c-d), demonstrating that CP induces the degradation of AcSGS3 via the autophagy pathway.

**Fig. 4:**
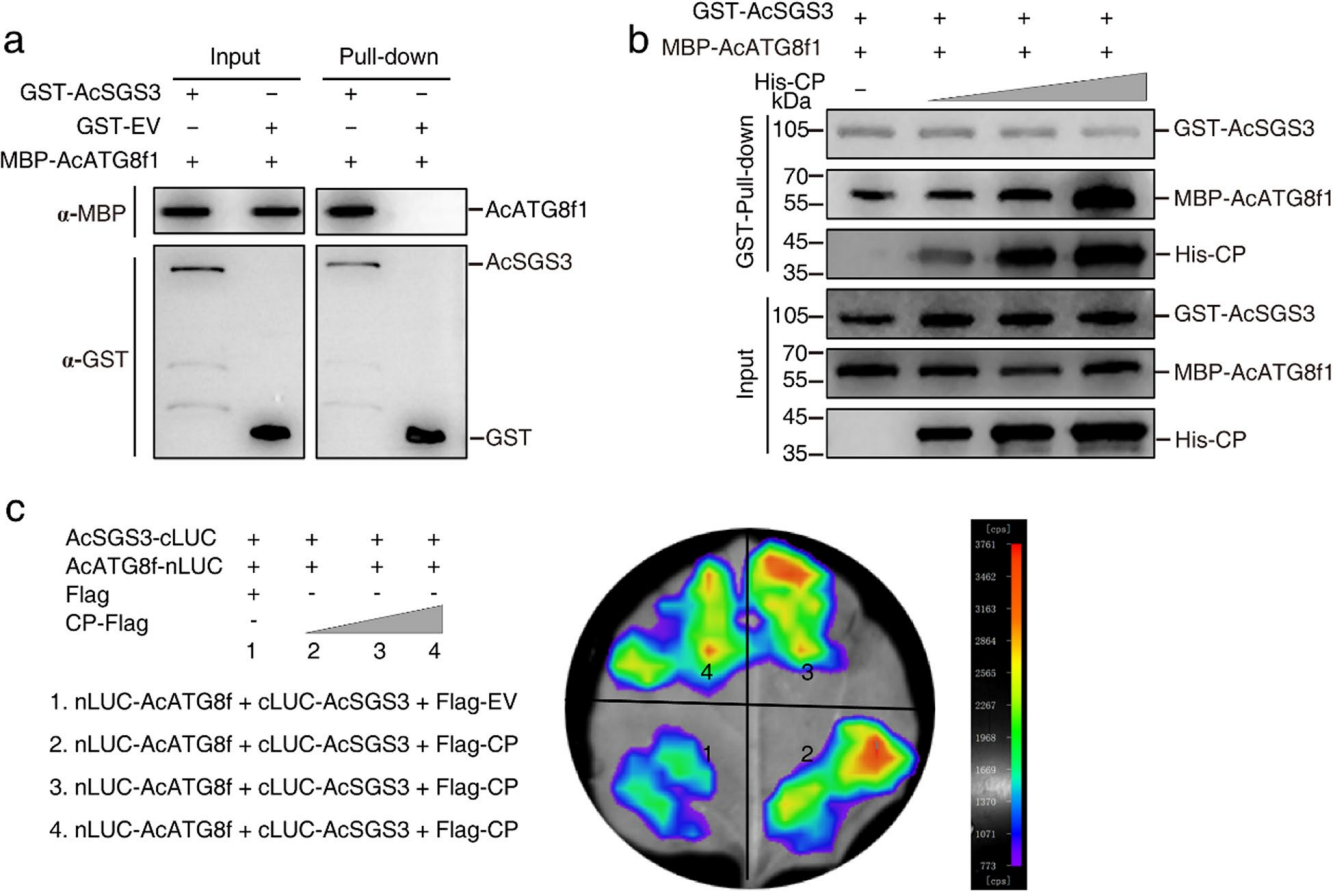
CP strengthens the interaction of autophagy related protein 8 (AcATG8) with AcSGS3. **a** Pull-down assay demonstrates the interaction of AcATG8 with AcSGS3. GST-AcSGS3 immobilized on Glutathione-Sepharose beads was incubated with MBP-AcATG8f1. Beads were washed and proteins were analyzed by Western-blot assays using anti-MBP (upper panel) and anti-GST (lower panel) antibodies, respectively. **b** The interaction between AcSGS3 and AcATG8f1 is disturbed by adding of CP in dose manner. GST-AcSGS3 and MBP-AcATG8f1 pull-down assay was added with increasing His-CP. Beads were washed and proteins were analyzed by Western-blot assays using anti-GST (upper panel), anti-MBP (middle panel) and anti-His (lower panel) antibodies, respectively. **c** Luciferase complementation assay (LCA) demonstrated CP enhances the interaction between AcSGS3 and AcATG8f1. cLuc-AcSGS3 and nLuc-AcATG8f1 were co-expressed in *N. benthamiana* leaves by agroinfiltration with gradually increasing CP-Flag. Luciferase activity was measured using the NightShade LB 985 In Vivo Plant Imaging System (Berthold Technologies, Germany) 72 hpi

Conjugation of ATG8 to the membrane lipid phosphatidylethanolamine (PE) is essential for autophagosome formation and cargo selection (Nishimura et al. 2017). Interaction with ATG8 is a hallmark of autophagic degradation. BiFC assays revealed interactions between AcATG8f1 and CP, as well as between AcATG8f1 and AcSGS3 (Fig. S4), which were further confirmed by Pull-down assays (Fig. 4a). Moreover, the interaction between AcATG8f1 and AcSGS3 was enhanced by CP in a dose-dependent manner (Fig. 4b), which was supported by LCA (Fig. 4c). These findings strongly demonstrate that CP mediates the degradation of SGS3 by promoting the interaction between AcSGS3 and AcATG8f1. Together, these results provide clear evidence that CP acts as a VSR by hijacking SGS3 to interact with ATG8 for autophagic degradation, representing a strategy employed by APV1 to suppress host RNA silencing.

### CP disrupts SGS3-RDR6 complex formation

**Fig. 5:**
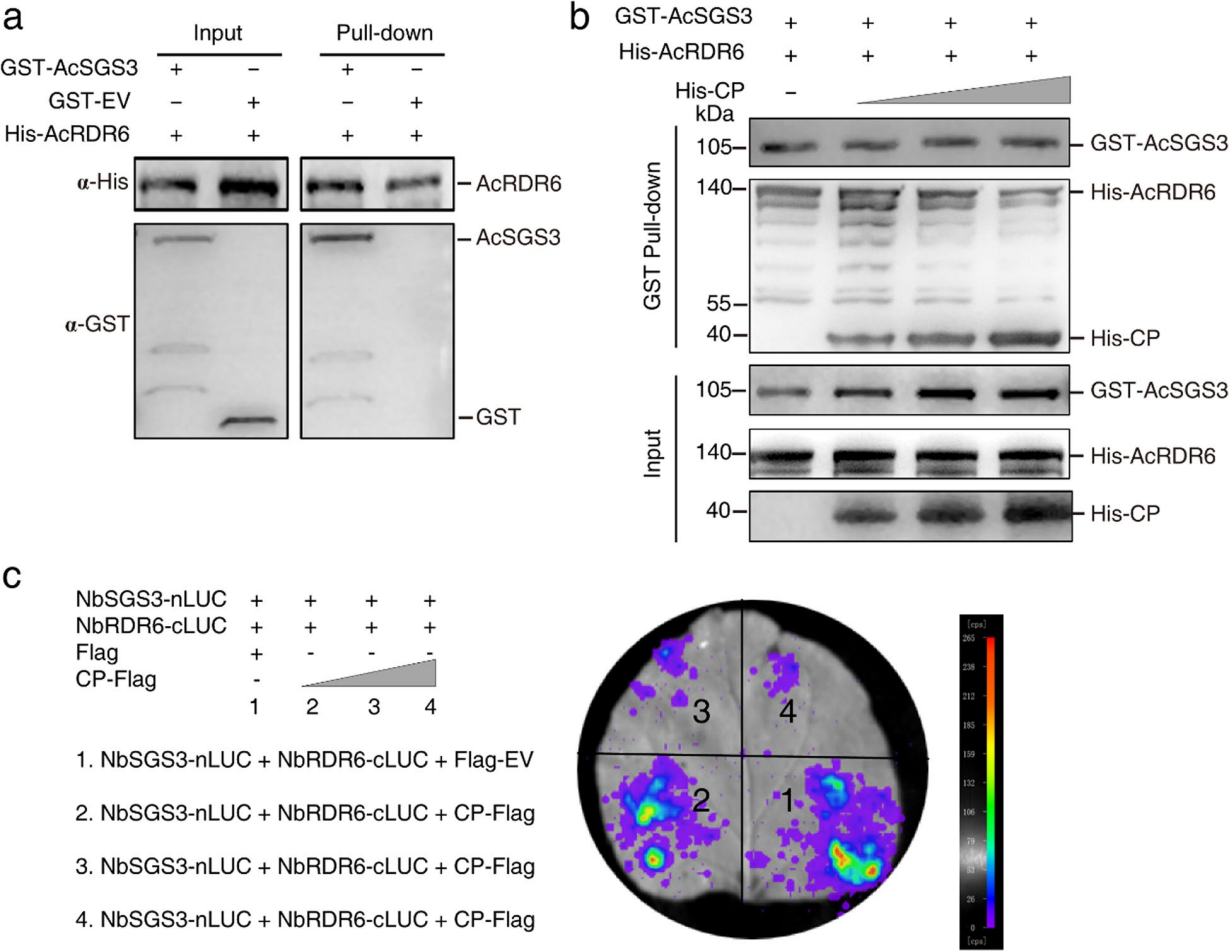
Assessing the impact of CP on the interaction between AcSGS3 and AcRDR6. **a** Pull-down assay demonstrates the in vitro interaction between AcSGS3 and AcRDR6. AcRDR6-6 × His protein immobilized on Pierce™ Ni–NTA Sepharose beads was incubated with *E. coli*-expressed recombinant GST-AcSGS3. Beads were washed and proteins were analyzed by Western-blot assays using anti-His (upper panel) antibodies and anti-GST (lower panel) antibodies, respectively. **b** The interaction between AcSGS3 and AcRDR6 is disturbed by adding of CP in dose manner. GST-AcSGS3 immobilized on glutathione-Sepharose beads was incubated with AcRDR6-6 × His protein and increasing His-CP. The eluted proteins were analyzed by Western-blot assays using anti-GST (upper panel), and anti-His antibodies (lower panel), respectively. **c** CP disrupts the interaction between NbSGS3 and NbRDR6 demonstrated by LCA. cLuc-NbSGS3 and nLuc-NbRDR6 were co-expressed in *N. benthamiana* leaves by agroinfiltration with gradually increasing CP-Flag. Luciferase activity was measured as Fig. 4c

Given that SGS3 forms functional complexes with RDR6 in cytoplasmic SGS3/RDR6 bodies to amplify RNA silencing signals, we investigated whether CP affects this critical interaction. Pull-down assays first confirmed the endogenous interaction between AcSGS3 and AcRDR6 from areca palm (Fig. 5a). Competitive binding experiments revealed that increasing concentrations of CP progressively disrupted the AcSGS3-AcRDR6 interaction (Fig. 5b). This observation was further supported by the LCA assays, which showed dose-dependent weakening of NbSGS3-NbRDR6 interaction in the presence of CP (Fig. 5c).

Summary, these findings collectively demonstrate that APV1-encoded CP employs a dual mechanism to suppress RNA silencing: (1) inducing autophagic degradation of AcSGS3, and (2) competitively disrupting the formation of SGS3-RDR6 silencing amplification complexes. CP act as a VSR through interfering with host autophagy and effectively dismantling both existing and nascent components of the host's RNA silencing machinery.

## Discussion

This study demonstrates that the CP of APV1 exploits host autophagy as a proviral mechanism to facilitate viral infection. Specifically, APV1 CP interacts with ATG8 to induce autophagy and promotes the formation of an ATG8–CP–SGS3 ternary complex. This complex enhances the association between SGS3 and ATG8, leading to the recruitment of SGS3 into autophagosomes and its subsequent degradation. Furthermore, CP disrupts the SGS3–RDR6 interaction, thereby suppressing the amplification of RNA silencing. These findings reveal a novel strategy by which APV1 CP harnesses autophagy to counteract antiviral RNA silencing, advancing our understanding of how closteroviruses manipulate host cellular pathways to promote infection.

The CP of closteroviruses is a multifunctional protein involved in virion assembly, viral movement, replication, and host–vector interactions (Weber and Bujarski 2015). Growing evidence supports its role as a viral VSR, as demonstrated in Citrus yellow vein clearing virus (CYVCV) (Ur Rehman et al. 2019; Zeng et al. 2023), Tomato chlorosis virus (Canizares et al. 2013), and CTV (Lu et al. 2004; Reed et al. 2003). Although CPs within the *Closteroviridae* family have been extensively studied, our work provides the first evidence that a closteroviral CP mediates autophagy-dependent degradation of SGS3 to counteract host defense.

VSRs typically interfere with host RNAi pathways by sequestering siRNA or dsRNA, or by disrupting key components of the silencing machinery. In plants, vsiRNAs are generated through DICER-like (DCL) processing of dsRNA, and some VSRs inhibit this step by suppressing DCL expression or activity (Azevedo et al. 2010). Closteroviruses often encode multiple VSRs that act at different stages of the silencing pathway (Lu et al. 2004; Reed et al. 2003). For instance, Tomato chlorosis virus employs both its CP and CPm as VSRs (Canizares et al., 2008), while CTV utilizes CP, p20, and p23 to inhibit RNA silencing (Lu et al. 2004). Although the mechanisms of many closteroviral VSRs remain unclear, recent studies indicate that they often target core silencing components. For example, Beet yellows virus p21 binds siRNAs to prevent their incorporation into RISC (Ye and Patel 2005), and CTV p20 recruits ATG8 to mediate autophagic degradation of SGS3 **(**Zhang et al. 2025**)**. SGS3, which forms a complex with RDR6, plays a critical role in the amplification of secondary siRNA production (Kumakura et al. 2009). A virus-induced small peptide 1 (VISP1) interacted with SGS3 and mediated autophagic degradation of SGS3/RDR6 bodies (Tong et al. 2021). APV1 encoded CP is the first VSR identified in *Velarivirus* genera.

To date, only a few members of the *Closteroviridae* family have been reported to modulate plant autophagy. For instance, the p22 protein of Tomato chlorosis virus (ToCV) interacts with NbBAG5 to inhibit autophagy and facilitate viral infection (Shang et al. 2023), though the functional role of autophagy during ToCV infection remains unknown. Another example is CTV, whose p20 protein recruits ATG8 to mediate autophagic degradation of SGS3, thereby suppressing RNA silencing (Zhang et al. 2025). The APV1 genome consists of a positive-sense single-stranded RNA of 17,419 nt. During viral replication, a long double-stranded RNA intermediate is likely produced and exposed in the plant cellular environment, which would inevitably be recognized by the host, triggering RNAi responses. Therefore, suppression of RNA silencing is essential for successful APV1 infection. In this study, we reveal that the CP of APV1 employs a novel dual mechanism to counteract host RNA silencing. On one hand, CP enhances the SGS3–ATG8 interaction and promotes the formation of an ATG8–CP–SGS3 ternary complex, leading to autophagic degradation of SGS3; on the other hand, it disrupts the SGS3–RDR6 complex and inhibits secondary siRNA amplification. This two-pronged strategy represents a previously unrecognized mode of action by which a closteroviral VSR suppresses antiviral silencing to promote viral infection.

The family *Closteroviridae* currently comprises seven genera and 57 species, as documented in the latest taxonomy release of the International Committee on Taxonomy of Viruses (ICTV). Many closteroviruses are pathogens of economically important crops, often leading to substantial yield losses (Dawson et al. 2013; Naidu et al. 2015; Folimonova and Sun 2022). Due to the general lack of natural resistance in host plants, conventional breeding for virus resistance remains challenging. As alternatives, virus-induced gene silencing (VIGS) and modern genome-editing technologies offer promising strategies for targeted modification of crop genomes to enhance virus resistance (Dawson and Folimonova 2013; Naidu et al. 2015; Folimonov et al. 2007). A critical step in this process is the identification of functional interaction sites between the viral protein and the host susceptibility factor. However, the presence of multiple interaction interfaces between CP and AcSGS3 complicates the selection of effective genomic editing targets. Characterization of the key residues for CP-SGS3 interaction still requires further investigation.

## Conclusions

This study identifies the APV1-encoded CP as a VSR. We demonstrate that CP interacts with the host AcSGS3, a critical factor in the RNA silencing pathway, and facilitates its degradation through autophagy. Furthermore, CP disrupts the association between AcSGS3 and AcRDR6, thereby interfering with the RNAi signaling cascade. These findings uncover a novel viral immune evasion mechanism in which APV1 CP hijacks the host autophagic machinery to degrade AcSGS3, effectively suppressing the plant’s antiviral RNA silencing defense.

## Materials and methods

### Plant materials

*N. benthamiana* seedlings (wild-type and GFP-transgenic line 16c) were germinated and grown in greenhouse under controlled conditions of 22 ± 2 °C, 16 h/8 h light/dark.

### Plasmid construction

All plasmids used in this study were constructed by using recombination-based one-step assembly (Vazyme, China). Full-length or individual domains of *AcSGS3* (GenBank accession PQ496828), *NbSGS3* (GenBank accession KJ190939) or *AcRDR6* (PX172336) were amplified by RT-PCR using specific primers (Table S1).

### Agroinfiltration and chemical treatments

The Agrobacterium infection procedure was performed as previous described (Li et al. 2017). For co-expression of two constructs, *A. tumefaciens* strains carrying each of the constructs were mixed right before infiltration in equal proportions (1:1) unless stated otherwise. Chemical inhibitors including MG132 (cat: HY-13259, MCE, Monmouth Junction, USA) and E64d (cat: HY-100229, MCE) were treated as previously described (Li et al. 2021).

### Protein extraction and Western blot assay

Total proteins from plant leaf samples were extracted using lysis buffer (50 mM Tris–HCl, pH 6.8, 4.5% SDS, 7.5% β-mercaptoethanol, 9 M urea). After centrifugation at 10,000 × g for 15 min at 4 °C, the supernatant was collected for Western blot assays. Proteins were detected using monoclonal antibodies against GFP (HT801; TransGen, Beijing, China), or His (1:5000; HT501; TransGen), or GST (HT601; TransGen), or Flag (R24091; ZEN-BIOSCIENCE, Chengdu, China). Horseradish peroxidase (HRP)-conjugated goat anti-mouse IgG (HS201; TransGen) or HRP-conjugated goat anti-rabbit IgG (AS014; ABclonal, Wuhan, China) were used as secondary antibodies. Blots were visualized with a chemiluminescence film (Thermo Fisher SuperSignal West Pico PLUS Chemiluminescent Substrate, catalog no. 34577).

### Protein induction and pull-down assays

The fusion proteins of GST-AcSGS3, GST-AcNTD, GSTAcZF, GST-AcXS, GST-AcCC, and His-AcRDR6 were expressed in *E. coli* BL21 (DE3) and purified using the GST-tagged or His-tagged protein purification kits (Beyotime, Shanghai, China, Cat #P2226 and Cat #P2262), respectively. Pull-down assays were conducted using Protein A/G MagBeads (GenScript, L00277; GenScript Biotech Corporation). The pull-down supernatant was collected for subsequent Western blot analysis.

### Bimolecular fluorescence complementation (BiFC) and confocal microscopy

For BiFC assays, *CP* CDS was cloned to fuse with the C-terminal YFP in binary vector pFGC-YC155 to produce YC-CP, while the full-length *AcSGS3* and each of its four domains were individually cloned to fuse with the N-terminal YFP in binary vector pFGC-YN173 to build YN-AcSGS3, YN-AcNTD, YN-AcZF, YN-AcXS, and YN-AcCC. The recombinant constructs were transiently expressed in *N. benthamiana* leaves via agroinfiltration. The epidermal cells of infiltrated leaves were examined for YFP fluorescence using the confocal laser scanning microscope LSM 780 (Carl Zeiss) with scan settings for YFP of Ex at 514 nm and Em at 565–585 nm. Images were processed with the LSM software (Carl Zeiss).

### Luciferase complementation assay (LCA)

CLA assays were performed as previously outlined (Cao et al. 2023), with constructs CP-cLUC and AcSGS3-nLUC. The nLUC/cLUC vector combinations were co-infiltrated into *N. benthamiana* leaves through agroinoculation. Leaves were harvested at 48 hpi, treated with 1 mM luciferin, and imaged using the NightShade LB 985 In Vivo Plant Imaging System (Berthold Technologies, Germany) two minutes after luciferin treatment.

## Supplementary Information

Supplementary Material 1.

## Data Availability

The data that support the findings of this study are available from the corresponding author upon reasonable request.

## Abbreviations

AcRDR6: RNA-dependent RNA polymerase 6 of *Areca catechu*
AcSGS3: Suppressor of Gene Silencing 3 of *Areca catechu*
APV1: Areca palm velarivirus 1
ATG8: Autophagy-related protein 8
BiFC: Bimolecular fluorescence complementation
CP: Coat protein
CPm: Minor coat protein
CTV: Citrus tristeza virus
DCL: DICER-LIKE proteins
LCA: Luciferase complementation assay
RNAi: RNA interfering
SGS3: Suppressor of Gene Silencing 3
VSR: Viral suppressor of RNA silencing
YLD: Yellow leaf disease
