# Supplementary material for: Areca palm velarivirus 1 encoded CP suppresses antiviral RNA silencing by mediating the autophagic degradation of SGS3 and disrupting the SGS3–RDR6 interaction
Source: Stress Biol. 2026 Jan 4;6(1):1. doi: 10.1007/s44154-025-00279-w (PMC12765794; doi:10.1007/s44154-025-00279-w)
Supplement: Supplementary file 1 — Supplementary Material 1. [file 44154_2025_279_MOESM1_ESM.docx]

**Supplemental materials**


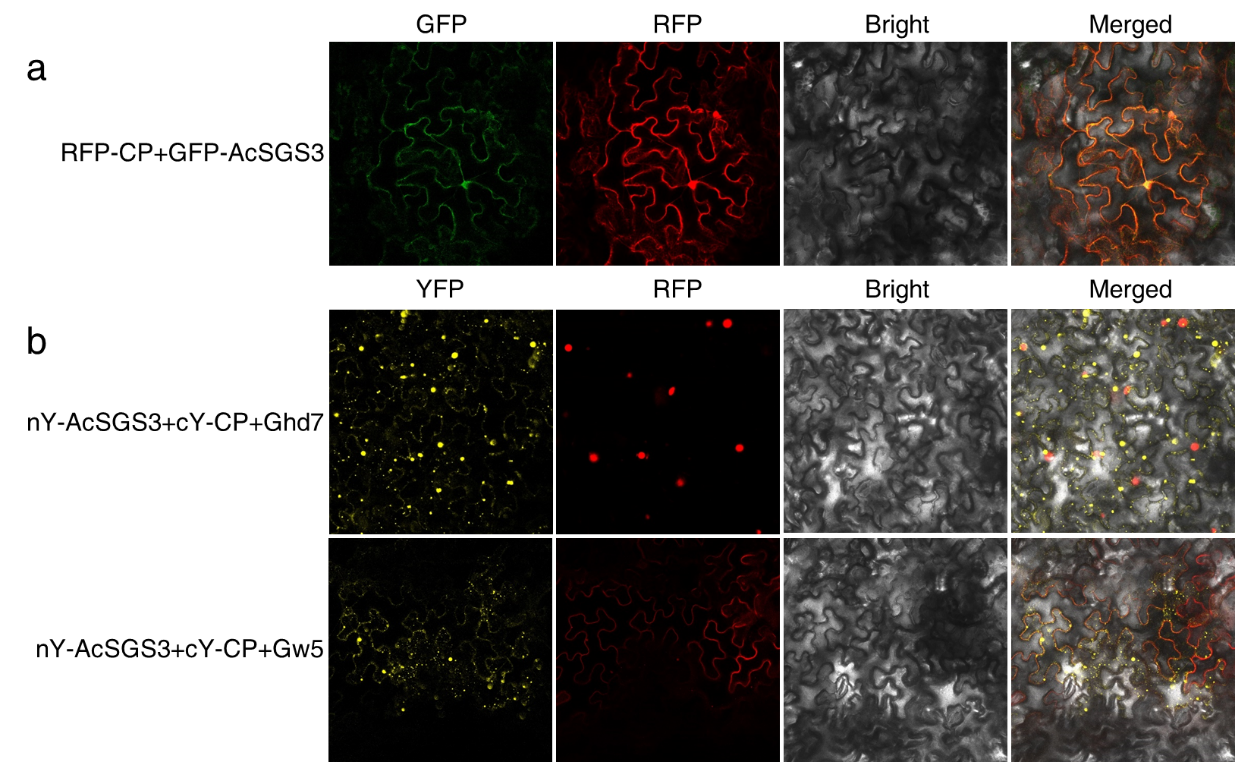


**Figure S1.**  **Subcellular localization of the interacted CP and AcSGS3.** **a.** REP-CP and GFP-AcSGS3 were transiently co-expressed in *N. benthamiana*. **b.** Bimolecular fluorescence complementation (BiFC) assay demonstrating the localization of the interacted complex of AcSGS3 and CP in the leaves of *N. benthamiana* at 72 h post-infiltration (hpi). The N- or C-terminal fragments of YFP were fused to the N-terminus of AcSGS3 and CP. Gw5-RFP and Ghd7-RFP were co-expressed respectively. The leaves were observed by confocal microscope at 72 hpi. Bars=50 μm.


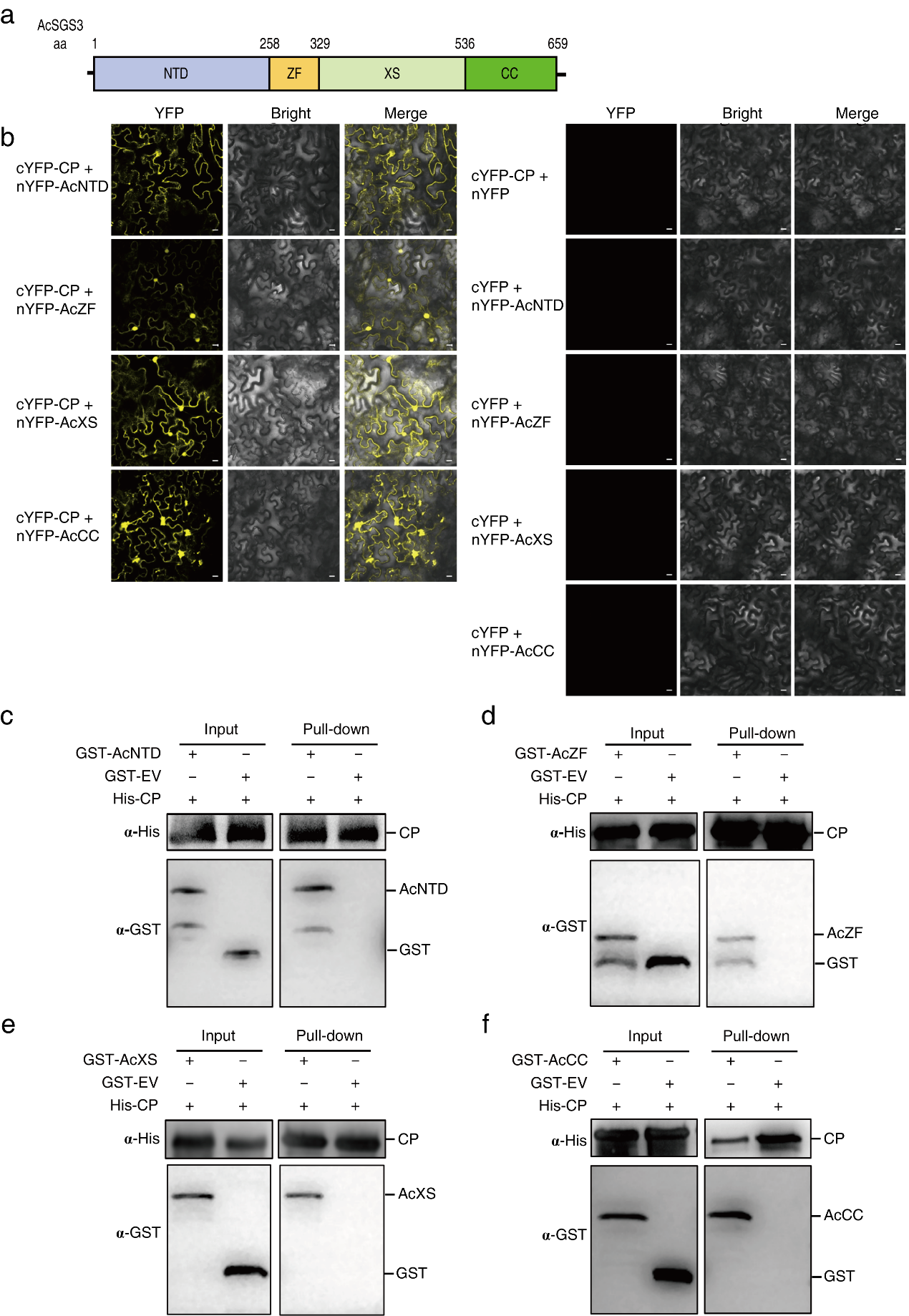


**Figure S2.** **Mapping of the CP-interacting domains in AcSGS3. a.** Schematic representation of the four predicted domains in AcSGS3. NTD, the N-terminal domain; ZF, the zinc finger domain; XS, the rice gene X and SGS3 domain; CC, the C-terminal coiled-coil domain. The numbers represent the amino acid positions of the right borders of the indicated domains (except for position 1 representing the left border of NTD). **b.** BiFC assays confirmed the interaction between CP and each of the four domains of AcSGS3. YFP fluorescence was visualized using confocal microscopy at 3 days post-infiltration (dpi). Scale bar=50 µm. **c–f.** Pull-down assays further validated the interactions between CP and each of the four domains of AcSGS3.


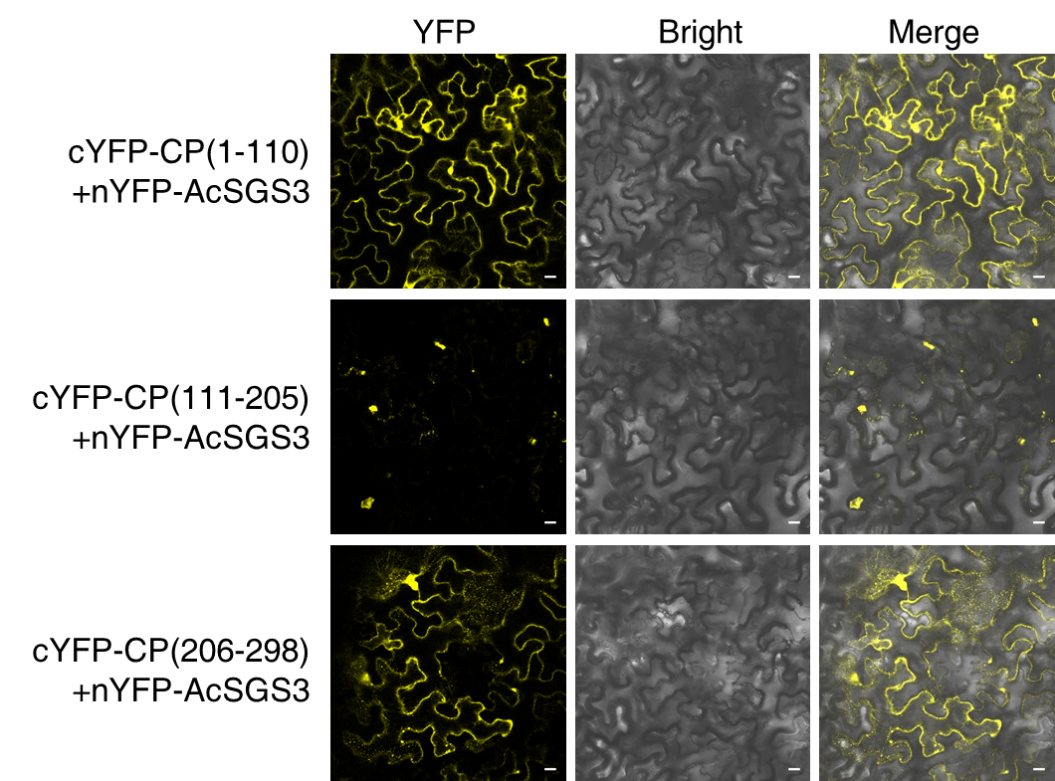


**Figure S3. Mapping of the AcSGS3-interacting domains in CP.** Bimolecular fluorescence complementation (BiFC) assay demonstrates the interaction between AcSGS3 fused to the N-terminal fragments of YFP and the truncated fragments (1-110aa, 111-205aa and 206-298aa) of CP fused with C-terminal of YFP respectively. Leaves of *N. benthamiana* was collected 72 h post-infiltration (hpi). Bars=20 μm.

**
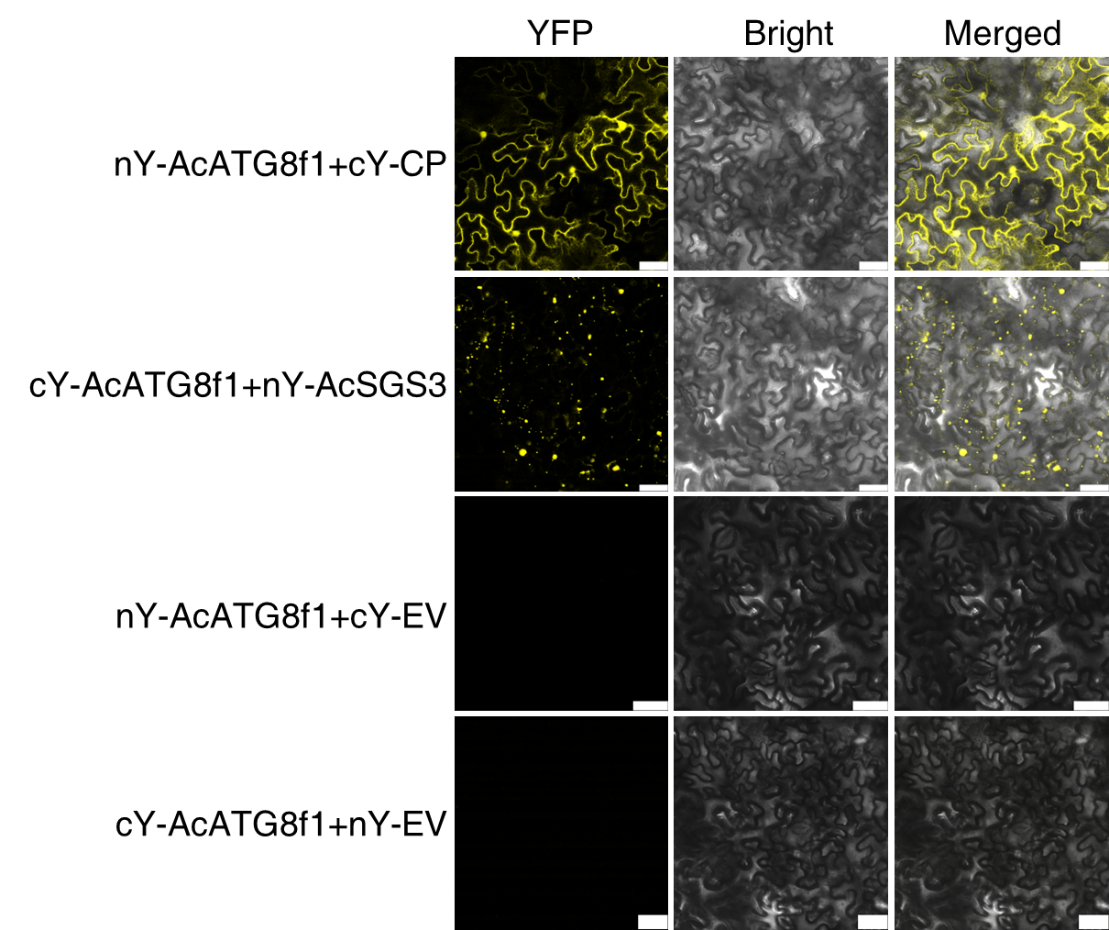
**

**Figure S4.** **Bimolecular fluorescence complementation (BiFC) demonstrates the interaction of autophagy related protein 8 (ATG8) with CP and with AcSGS3.** The combinations of nYFP-AcATG8f1 and cYFP-CP, cYFP-AcATG8f1 and cYFP-SGS3, cYFP-AcATG8f1 and cYFP-CP, cYFP-AcATG8f1 and nYFP-vector, and nYFP-AcATG8f1 and cYFP-vector were transiently co-expressed in the leaves of *N. benthamiana,* respectively*.* The leaves were observed by confocal microscope at 72 h post-infiltration (hpi). Bars=50 μm.

**Table S1.** **DNA primers used in the study.**

| Primer name | Primer sequence (5’-3’) | Note |
| --- | --- | --- |
| Y-Flag-opt-CP-F | GAGCTCGGTACCCGGGGATCCATGGACCGTTTGAACCTGCT | construction of p35S-Flag-CP |
| Y-Flag-opt-CP-R | ATGGTCTTTGTAGTCGTCGACCCTGCGAGTATTACCCACAA |  |
| Y-Flag-TBSV-p19-F | GAGAACACGGGGGACGAGCTCATGGAACGAGCTATACAAG | construction of p35S-Flag-p19 |
| Y-Flag-TBSV-p19-R | TCGTCGACTCTAGAGGATCCCTCGCTTTCTTTTTCGAAGG |  |
| Y-GFP-AcSGS3-F | GAGAACACGGGGGACGAGCTCATGAATTTAAGAAAGGGT | construction of p35S-GFP-AcSGS3 |
| Y-GFP-AcSGS3-R | CATGTCGACTCTAGAGGATCCAGAGCTGCTGGTTGAAGC |  |
| Y-RFP-CP-F | GAGAACACGGGGGACGAGCTCATGGATAGACTTAACTTACTT | construction of p35S-RFP-CP |
| Y-RFP-CP-R | CATGTCGACTCTAGAGGATCCCCGTCTAGTATTACCAACAATT |  |
| cY-CP-F | CTCTCGAGCTTTCGCGAGCTCATGGATAGACTTAACTTACT | construction of YC-CP |
| cY-CP-R | CGCCGGACGGGTACCGGATCCCCGTCTAGTATTACCAACAATTTG |  |
| nY-NbSGS3-F | CTCTCGAGCTTTCGCGAGCTCatgagttcaagcaaaggg | construction of YN-NbSGS3 |
| nY-NbSGS3-R | GATGGATCTTCTAGAGGATCCttgagattgctctggggagt |  |
| nY-AcSGS3-F | CTCTCGAGCTTTCGCGAGCTCATGAATTTAAGAAAGGGT | construction of YN-AcSGS3 |
| nY-AcSGS3-R | GATGGATCTTCTAGAGGATCCAGAGCTGCTGGTTGAAGCCT |  |
| nY-AcNTD-F | CTCTCGAGCTTTCGCGAGCTCATGAATTTAAGAAAGGGT | construction of YN-AcNTD |
| nY-AcNTD-R | GATGGATCTTCTAGAGGATCCGTGCCACTGTCTTGTTTGT |  |
| nY-AcZF-F | CTCTCGAGCTTTCGCGAGCTCATGTGCCCGGCATGTCAT | construction of YN-AcZF |
| nY-AcZF-R | GATGGATCTTCTAGAGGATCCAGTGGTTGTCTCCCGAAGA |  |
| nY-AcXS-F | CTCTCGAGCTTTCGCGAGCTCATGGATCATTTGACAGTT | construction of YN-AcXS |
| nY-AcXS-R | GATGGATCTTCTAGAGGATCCGTAATCCATCTCTTCTTT |  |
| nY-AcCC-F | CTCTCGAGCTTTCGCGAGCTCATGCAAGAGCGATTTTT | construction of YN-AcCC |
| nY-AcCC-R | GATGGATCTTCTAGAGGATCCAGAGCTGCTGGTTGAAGCCT |  |
| cY-Acatg8f1(366)-F | CTCTCGAGCTTTCGCGAGCTCATGGCGAGGAGTTCCTTCA | construction of YC-AcATG8f1 |
| cY-AcATG8f1-366-R | CGCCGGACGGGTACCGGATCCGACAAGAATCTCAAGCCCA |  |
| nY-Acatg8f1(366)-F | CTCTCGAGCTTTCGCGAGCTCATGGCGAGGAGTTCCTTCA | construction of YN-AcATG8f1 |
| nY-Acatg8f1(366)-R | GATGGATCTTCTAGAGGATCCGACAAGAATCTCAAGCCCA |  |
| cY-CP(1-110）-R | CGCCGGACGGGTACCGGATCCTTGACAAACGAAATCAAC | Construction of truncated mutants of cY-CP |
| cY-CP(111-205)-F | CTCTCGAGCTTTCGCGAGCTCATGGCGCCGGATGAACTA |  |
| cY-CP(111-205)-R | CGCCGGACGGGTACCGGATCCTGCCTTAAGTGCATTGACC |  |
| cY-CP(206-298)-F | CTCTCGAGCTTTCGCGAGCTCatgATTCCCAACGTGTTGC |  |
| CLUC-OPT-ORF6-F | CCGGGGCGGTACCcgggatccaGACCGTTTGAACCTGCTC | construction of cLUC-CP |
| CLUC-OPT-ORF6-R | CGAAAGCTCTGCAGGTCGACCTACCTGCGAGTATTACCCA |  |
| nLUC-AcSGS3-F | gagctcggtacccgggatccATGAATTTAAGAAAGGGTGGA | construction of nLUC-AcSGS3 |
| nLUC-AcSGS3-R | cgcgtacgagatctggtcgacAGAGCTGCTGGTTGAAGCCTC |  |
| nLUC-NbSGS3-F | gagctcggtacccgggatccatgagttcaagcaaaggggt | construction of nLUC-NbSGS3 |
| nLUC-NbSGS3-R | acgcgtacgagatctggtcgacttgagattgctctggggagt |  |
| cLUC-AcSGS3-F | CCGGGGCGGTACCcgggatccaATGAATTTAAGAAAGGGTGGA | construction of cLUC-AcSGS3 |
| cLUC-AcSGS3-R | ACGAAAGCTCTGCAGGTCGACCTAAGAGCTGCTGGTTGAAGC |  |
| cLUC-NbRDR6-F | GGGCGGTACCcgggatccaGGATCAGAGGGCTCTGAAAA | construction of cLUC-NbRDR6 |
| cLUC-NbRDR6-R | ACGAAAGCTCTGCAGGTCGACCTATATATTGTCAACAAGATACCTTC |  |
| nLUC-AcATG8f1(366)-F | GCTCGGTACCcgggatccATGGCGAGGAGTTCCTTCAA | construction of nLUC-AcATG8f1 |
| nLUC-AcATG8f1(366)-R | cgtacgagatctggtcgacGACAAGAATCTCAAGCCCAA |  |
| GST-AcSGS3-F | CCCCTGGGATCCCCGGAATTCAATTTAAGAAAGGGTGGA | construction of GST-AcSGS3 |
| GST-AcSGS3-R | GATGCGGCCGCTCGAGTCGACCTAAGAGCTGCTGGTTGAA |  |
| his-CP-F | GCCATGGCTGATATCGGATCCATGGATAGACTTAACTTAC | construction of His-CP |
| his-CP-R | GTGGTGGTGGTGGTGCTCGAGTTACCGTCTAGTATTACCA |  |
| His-AcRDR6-F | ACAGCAAATGGGTCGGGATCCGATGGGTTCGCTAGGGGCA | construction of His-AcRDR6 |
| His-AcRDR6-R | CTCGAGTGCGGCCGCAAGCTTTATCCTTTCCGAAAGATAGCG |  |
| GST-AcNTD-F | CCTGGGATCCCCGGAATTCATGAATTTAAGAAAGGGTGG | construction of GST-AcNTD |
| GST-AcNTD-R | GCGGCCGCTCGAGTCGACCTAGTGCCACTGTCTTGTTTGT |  |
| GST-AcZF-F | CCCCTGGGATCCCCGGAATTCTGCCCGGCATGTCATAATG | construction of GST-AcZF |
| GST-AcZF-R | GCGGCCGCTCGAGTCGACCTAAGTGGTTGTCTCCCGAAG |  |
| GST-AcXS-F | CCCTGGGATCCCCGGAATTCGATCATTTGACAGTTTGGCC | construction of GST-AcXS |
| GST-AcXS-R | CGGCCGCTCGAGTCGACCTAGTAATCCATCTCTTCTTTATTC |  |
| GST-AcCC-F | CCCTGGGATCCCCGGAATTCCAAGAGCGATTTTTCAAGGAG | construction of GST-AcCC |
| GST-AcCC-R | GCGGCCGCTCGAGTCGACCTAAGAGCTGCTGGTTGAAG |  |
| MBP-AcATG8f1-366-F | AGGATTTCAGAATTCGGATCCATGGCGAGGAGTTCCTTC | construction of MBP-AcATG8f1 |
| MBP-AcATG8f1-366-R | ACGGCCAGTGCCAAGCTTCTAGACAAGAATCTCAAGCCC |  |
